# Susceptibility to Neurodegeneration in a Glaucoma Is Modified by *Bax* Gene Dosage

**DOI:** 10.1371/journal.pgen.0010004

**Published:** 2005-07-25

**Authors:** Richard T Libby, Yan Li, Olga V Savinova, Joseph Barter, Richard S Smith, Robert W Nickells, Simon W.M John

**Affiliations:** 1 The Jackson Laboratory, Bar Harbor, Maine, United States of America; 2 Department of Ophthalmology and Visual Sciences, University of Wisconsin, Madison, Wisconsin, United States of America; 3 The Howard Hughes Medical Institute, Bar Harbor, Maine, United States of America; 4 Department of Ophthalmology, Tufts University School of Medicine, Boston, Massachusetts, United States of America; Johns Hopkins Institute, United States of America

## Abstract

In glaucoma, harmful intraocular pressure often contributes to retinal ganglion cell death. It is not clear, however, if intraocular pressure directly insults the retinal ganglion cell axon, the soma, or both. The pathways that mediate pressure-induced retinal ganglion cell death are poorly defined, and no molecules are known to be required. DBA/2J mice deficient in the proapoptotic molecule BCL2-associated X protein (BAX) were used to investigate the roles of BAX-mediated cell death pathways in glaucoma. Both *Bax* ^+/−^ and *Bax* ^−/−^ mice were protected from retinal ganglion cell death. In contrast, axonal degeneration was not prevented in either *Bax* ^+/−^ or *Bax* ^−/−^ mice. While BAX deficiency did not prevent axonal degeneration, it did slow axonal loss. Additionally, we compared the effects of BAX deficiency on the glaucoma to its effects on retinal ganglion cell death due to two insults that are proposed to participate in glaucoma. As in the glaucoma, BAX deficiency protected retinal ganglion cells after axon injury by optic nerve crush. However, it did not protect retinal ganglion cells from *N*-methyl-D-aspartate (NMDA)-induced excitotoxicity. BAX is required for retinal ganglion cell death in an inherited glaucoma; however, it is not required for retinal ganglion cell axon degeneration. This indicates that distinct somal and axonal degeneration pathways are active in this glaucoma. Finally, our data support a role for optic nerve injury but not for NMDA receptor-mediated excitotoxicity in this glaucoma. These findings indicate a need to understand axon-specific degeneration pathways in glaucoma, and they suggest that distinct somal and axonal degeneration pathways may need to be targeted to save vision.

## Synopsis

Glaucoma is a group of diseases whose unifying characteristic is death of nerve cells (retinal ganglion cells) that connect the eye to the brain. Glaucoma is often associated with a harmfully high pressure inside the eye (intraocular pressure) contributing to nerve cell death. Various treatments are used to lower eye pressure, but currently no commonly used treatments directly protect the nerve cells. DBA/2J mice develop elevated eye pressure with age, and this pressure kills retinal nerve cells. The authors use this mouse model to investigate how these nerve cells die in glaucoma. They show that there are distinct degeneration pathways activated in different parts of the retinal nerve cells. They found that the biochemical pathway in the nerve cell body, which resides in the retina, requires a molecule called BAX (BCL2-associated X protein). In contrast, pathways in the part of the cell (axon) that connects the cell body to the brain do not require BAX. Because degeneration pathways in the cell body and of the axon also may be molecularly different in human glaucoma, it will be important to consider them all when designing therapies. Their data also suggest that the BAX gene is a candidate to modulate glaucoma susceptibility.

## Introduction

Glaucoma is a common blinding disease affecting approximately 70 million people worldwide [1]. Glaucoma is often associated with elevated intraocular pressure (IOP). IOP elevation and glaucoma are typically spontaneous, progressive, idiopathic processes and are most common in the elderly [2]. Although IOP-lowering treatments slow the development and progression of glaucoma in many patients [3,4], it is not always possible to reduce IOP to a “safe” level [5]. Vision loss in glaucoma is the result of retinal ganglion cell (RGC) death with accompanying optic nerve atrophy, so glaucoma is a neuropathy. IOP elevation is not detected in a significant subset of glaucomas [6,7]. Thus, the unifying characteristic of glaucoma is RGC death. While there are several hypotheses as to why elevated IOP kills RGCs, both the precise biochemical cascades that are triggered within RGCs and the nature of the proximal insult(s) that trigger these cascades remain superficially defined [8]. No treatments that directly protect the neurons are in routine clinical use.

The complex nature of glaucoma makes studies of its pathogenesis difficult [9]. Consequently, no specific molecules have been shown to be essential for RGC death in glaucoma. Standard glaucoma-relevant models include direct RGC trauma, direct optic nerve trauma, and suddenly induced IOP elevation [10–18]. Although these induced models have provided valuable information, the relevance of specific damaging mechanisms may differ significantly between spontaneous and experimentally induced glaucomas. Thus, studies using inherited glaucoma models are also necessary.

Apoptosis is known to contribute to RGC death following experimentally induced insults including axotomy and IOP elevation (e.g.*,* [19,20]), and there is also some evidence that apoptosis is involved in human glaucoma [21,22]. A number of molecules that are known to affect apoptosis are reported to be important regulators of RGC death after various induced insults. These include X-linked inhibitor of apoptosis protein (XIAP) [23–25], p38 [26], several caspases [27–30], the B-cell lymphoma/leukemia 2 (BCL2) family of apoptotic regulators [20,31–34], and members of the c-Jun N-terminal kinase (JNK) [35,36] and tumor necrosis factor (TNF) [35,37] signaling pathways. One of these molecules, BCL2-associated X protein (BAX; a proapoptotic member of the BCL2 family), has a major role in mitochondrial-mediated apoptosis in different neuronal cell types [38,39]. In mice, BAX deficiency increases the number of RGCs in the adult retina by 220% by allowing more RGCs to survive during development [39]. Genetic or induced BAX deficiency is also known to prevent RGC apoptosis after optic nerve crush and axotomy [13,18,40]. Thus, BAX-mediated apoptosis is clearly an important mechanism of stress-induced RGC death. Whether or not this pathway has a role in IOP-induced RGC death in either experimentally induced or inherited glaucomas is not known.

Understanding the pathophysiologic mechanisms of RGC death in glaucoma and the genetic susceptibility factors contributing to this process is important for the development of effective and individualized treatments. Here, we use the genetically uniform DBA/2J mouse model of glaucoma [41–43] to assess the importance of mitochondrially mediated apoptosis in an inherited glaucoma. Importantly, we show that in this model of inherited glaucoma there are distinct RGC death and axonal degeneration pathways. The RGC death pathway is BAX dependent and, therefore, apoptotic. The axonal degeneration pathway is BAX independent. Finally, our data suggest that reducing BAX levels in the retina may retard the rate of vision loss in glaucoma.

## Results

### Apoptosis Is Physiologically Relevant for RGC Death in an Inherited Glaucoma

**Figure 1:**
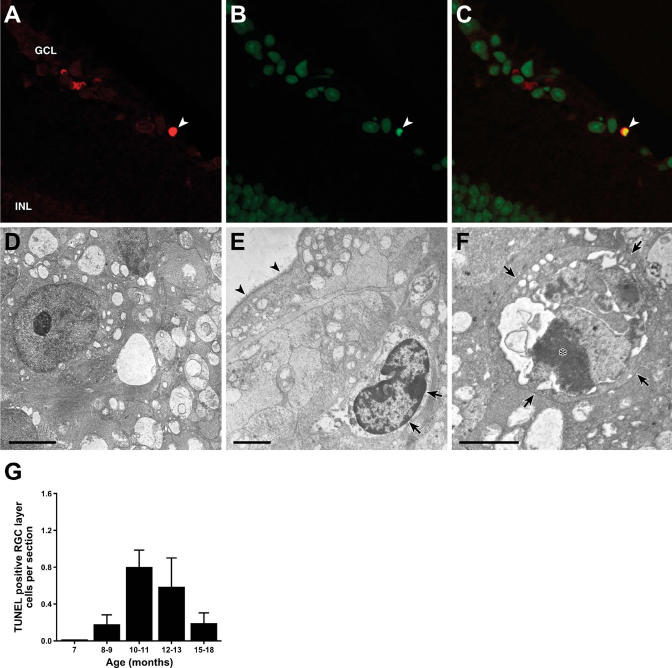
Dying RGCs Have Characteristic Features of Apoptosis (A–C) A double-labeling assay that identifies fragmented DNA using fluorescently labeled dUTP (A) and detects chromatin condensation by binding of the dye YOYO-1 (B) was used to assess the presence of these hallmarks of apoptosis in glaucomatous DBA/2J eyes at 10–11 mo of age (a time when many RGCs die). A cell in the retinal ganglion cell layer (GCL, arrowhead) has both of these features of apoptosis as indicated by double labeling (C). INL, inner nuclear layer. (D–F) Electron microscopy provided further evidence for apoptosis. (D) An example of a healthy RGC. (E) Chromatin condensation (a hallmark of apoptosis) along the inner surface of the nuclear envelope in a ganglion cell (arrows). The internal limiting membrane of the retina is indicated by arrowheads. (F) An apoptotic body in the ganglion cell layer (arrows) containing a nuclear fragment with prominent condensed chromatin (asterisk) and other cell remnants. (G) A TUNEL assay (see Materials and Methods) was used to assess the prevalence of cell death at different ages. TUNEL labeling was not detected at 7 mo (an age prior to glaucomatous cell death) and peaked at 10–13 mo, when most RGCs die. No TUNEL-positive cells were detected in nonglaucomatous, age-matched control mice. These results support an important role of apoptosis in RGC death in spontaneous glaucoma. Scale bar, 1 μm.

To determine if RGC apoptosis has a significant role in an inherited glaucoma, we assessed DNA fragmentation, chromatin condensation, and cellular ultrastructure in glaucomatous DBA/2J retinas. We identified hallmarks of apoptosis including the presence of TUNEL-positive cells that peaked between 10 and 13 mo of age (the period when the majority of RGC death occurs in this model) (Figure 1). These results confirm earlier suggestive studies that apoptotic pathways are important mediators of RGC death in spontaneous glaucoma [19–22].

### Homozygous but Not Heterozygous BAX Deficiency Alters RGC Number

To test the role of BAX in glaucomatous RGC death, we extensively backcrossed a previously characterized null allele of *Bax* *(Bax^tm1Sjk^)* [44] onto the inbred DBA/2J background. In mammals, approximately twice as many RGCs are produced during retinal development than survive into adulthood [45–47]. As expected from previous studies of retinal development on a different genetic background [39], complete BAX deficiency increased the number of RGC-layer somata in adult DBA/2J mice by 220% (average cell number per 40× field ± standard error of the mean [SEM], number of retinas analyzed: *Bax* ^+/+^, 199 ± 5.6, *n* = 7; *Bax* ^−/−^, 437 ± 15.3, *n* = 8). In agreement with this, *Bax* ^−/−^ mice had 217% more RGC axons than *Bax* ^+/+^ mice (*Bax* ^+/+^, 50,504 ± 1,988, *n* = 8; *Bax* ^−/−^, 108,907 ± 10,322, *n* = 4; *p* < 0.001). Reflecting the increased number of RGC axons and the proportional increase in glial cell types [48], the cross sectional area of *Bax* ^−/−^ optic nerves was significantly increased (average ± SEM, number of optic nerves measured: *Bax* ^+/+^, 0.157 ± 0.005 mm^2^, *n* = 13; *Bax* ^−/−^, 0.278 ± 0.008 mm^2^, *n* = 16; *p* < 0.001). In heterozygous *Bax* ^+/−^ mice, RGC number (average per 40× field ± SEM, 212 ± 14.0, *n* =5) and the optic nerve area (0.171 ± 0.007 mm^2^, *n* = 13) was not different from *Bax* ^+/+^ mice (*p* = 0.352 and *p* = 0.107, respectively). Thus, heterozygous levels of BAX are sufficient for death of the normal numbers of RGCs during retinal development.

### BAX Ablation Preserves RGC Numbers but Does Not Prevent RGC Axonal Degeneration in Glaucoma

**Figure 2:**
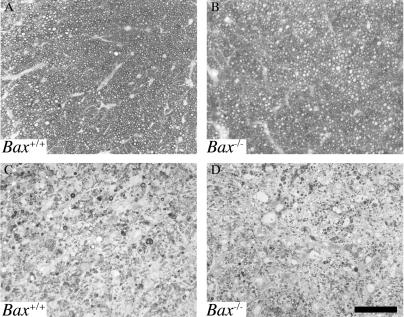
BAX Is Not Required for Glaucomatous Optic Nerve Degeneration To assess the effects of *Bax* deficiency on optic nerve degeneration, we analyzed PPD-stained optic nerve cross sections from *Bax* ^+/+^ and *Bax* ^−/−^ mice (*n* > 49 for each genotype; see Materials and Methods). (A and B) Before the DBA/2J glaucoma damages RGCs, the optic nerves of both *Bax* ^+/+^ (A) and *Bax* ^−/−^ mice (B) had a normal organization. The axons appeared healthy with a clear axoplasm and darkly stained myelin sheath. (C and D) BAX deficiency did not prevent glaucomatous optic nerve damage. Severe degeneration involving extensive to complete axon loss and scarring occurred in both *Bax* ^+/+^ (C) and *Bax* ^−/−^ mice (D). The majority of mice of both genotypes had this severe degree of damage by 12 mo of age. These experiments show that BAX is not required for glaucomatous axon degeneration. Scale bar, 50 μm.

**Figure 3:**
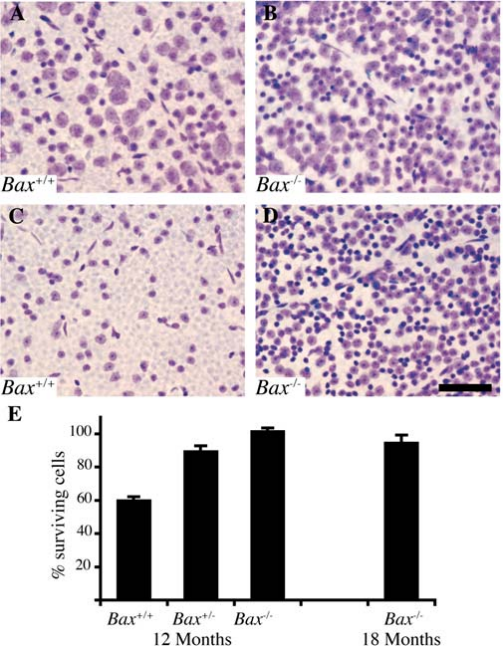
*Bax* Deficiency Prevents Glaucomatous RGC Death To determine the effects of BAX deficiency on RGC death in glaucoma, we analyzed RGC layer cells at stages with and without glaucomatous optic nerve damage (see Materials and Methods). All shown images are from a similar region of the superior, peripheral retina. (A and B) In both *Bax^+/+^* (A) and *Bax* ^−/−^ (B) mice without glaucomatous optic nerve damage, the retinas appear healthy. The retinas of both genotypes are similar except that *Bax* ^−/−^ mice have extra RGCs (since BAX is important in normal developmental RGC death [39]). (C and D) In contrast, an obvious difference was evident between the retinas of *Bax* ^+/+^ and *Bax* ^−/−^ mice that had all suffered severe glaucomatous damage with 95% or more axon degeneration. As expected, for *Bax* ^+/+^ retinas (C) from eyes with 95% or more optic nerve axon loss, there was a noticeable decrease in RGC layer cells (compare [C] to [A]). In contrast, retinas from *Bax* ^−/−^ mice with correspondingly damaged optic nerves (D) had suffered no obvious loss of RGC layer cells (compare [D] to [B]). This suggests that BAX is required for RGC death in DBA/2J glaucoma. As is well established for both RGCs and other neurons, *Bax* ^−/−^ RGCs that survive without axons have a shrunken morphology [38,82]. This is clearly evident in the *Bax* ^−/−^ glaucomatous mice (D). (E) RGC layer cell counts for eyes with 95% or more axon degeneration confirmed that BAX is necessary for RGC death in this glaucoma. To allow comparison between genotypes, the percent of surviving cells is shown (% soma in mice with 95% or more axon loss compared to mice of the same genotype without glaucomatous damage). At 12 mo of age, *Bax* ^+/+^ mice had 61.4% ± 3.8% of their RGC layer cells remaining, *Bax* ^−/−^ mice had no appreciable cell loss (101% ± 5.3%). The RGC layer cells of *Bax* ^+/−^ mice were also protected (89.2% ± 5.7%). The *p* values comparing differences in cell counts between nonglaucomatous and very severely glaucomatous (≥ 95% axon loss) eyes of the same genotype were: *Bax* ^+/+^, *p* < 0.001; *Bax* ^+/−^, *p* = 0.207; *Bax* ^−/−^, *p* = 0.426. No cell loss was seen in *Bax* ^−/−^ mice even out to 18 mo (94.8% ± 4.4% cells surviving, *p* = 0.524 compared to nonglaucomatous *Bax* ^−/−^ mice). These findings show that BAX gene dosage has an important effect on the susceptibility of RGCs to glaucomatous death. Scale bar, 50 μm.

To determine the role of BAX in glaucomatous RGC death, we assessed the effects of BAX deficiency on RGC survival and on RGC axonal degeneration (see Materials and Methods). Our results show that BAX is not required for RGC axon degeneration. *Bax* ^−/−^ mice developed severe optic nerve damage, including essentially complete loss of axons (Figure 2). In contrast, our experiments show that BAX is required for RGC death in glaucoma (Figure 3). Despite severe axonal degeneration, the numbers of cell bodies in the RGC layer of *Bax* ^−/−^ mice were normal. As a stringent test of this observation, we counted RGC-layer cell bodies in the retinas of mice with severe (≥ 95% loss) axon degeneration. The number of RGC cell bodies was normal in *Bax* ^−/−^ mice with more than 95% axon loss (Figure 3). Importantly, *Bax* ^+/−^ mice were also protected against glaucomatous RGC death. *Bax* ^+/−^ mice with an axon loss of 95% or more also had substantially increased survival of RGC cell bodies as compared to *Bax* ^+/+^ controls (Figure 3E). Thus, RGC death and axonal degeneration are clearly distinguished in these experiments.

### Other Proapoptotic Molecules Do Not Compensate for BAX Deficiency

In some neuronal cell types, BAX deficiency delays but does not prevent apoptosis [49]. This is because other proapoptotic molecules (e.g., another BCL2 family member, BAK) mediate cell death in the BAX-deficient neurons [50]. To test this possibility in the DBA/2J model, we aged *Bax* ^−/−^ mice to18 mo. As expected in a complex age-related disease, the severity of glaucomatous damage varies between individual DBA/2J eyes at any age. Nevertheless, by 12 mo of age, the majority of eyes have severe optic nerve damage (see below). Therefore, 18 mo of age is 6 mo after the majority of eyes have severe axon loss. Despite this extensive axonal degeneration, there was no obvious reduction in RGC numbers in any of the 18-mo-old *Bax* ^−/−^ eyes (Figure 3E). This result indicates that other molecules do not substitute for BAX and that BAX is essential for RGC apoptosis in DBA/2J inherited glaucoma.

### Homozygous BAX Deficiency Alters IOP

DBA/2J mice develop a form of pigmentary glaucoma that is secondary to a progressive iris disease. Iris pigment and cell debris enter the ocular drainage structures, resulting in subsequent IOP elevation [41,42]. The increase in IOP induces RGC death. Manipulations that alleviate the iris disease and prevent IOP elevation also prevent RGC death in this strain [41]. To assess the effects of BAX deficiency, the clinical phenotypes and IOP profiles of mice of each *Bax* genotype were carefully examined at multiple ages.

Periodic assessment of the progression of iris abnormalities by slit-lamp examinations (approximately every 2 mo between 3 and 12 mo of age) revealed no differences between mice of each *Bax* genotype. Histologic analysis confirmed these observations (unpublished data). Thus, BAX-mediated processes are not necessary for the progression of the iris disease.

**Figure 4:**
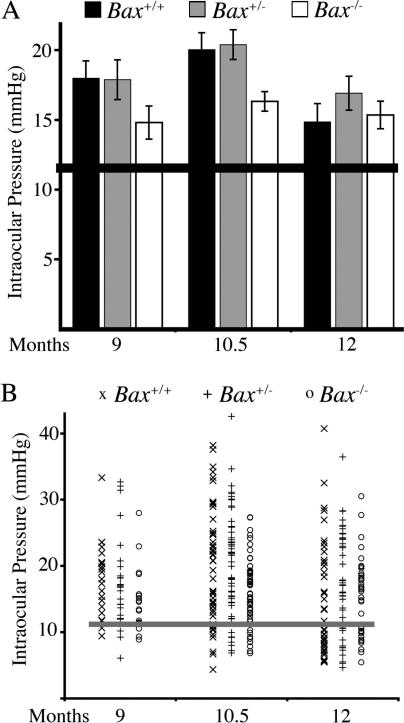
Complete *Bax* Deficiency Has a Protective Effect against IOP Elevation To assess the possibility that *Bax* deficiency may delay axon degeneration by lessening the glaucomatous insult to which RGCs are exposed, we analyzed IOP at key ages of IOP elevation. (A) The average IOP for each genotype (± SEM) and (B) the actual IOP values recorded. *Bax* deficiency did not prevent IOP elevation. At both 9 mo and 10.5 mo, the average IOP of both *Bax* ^+/−^ and *Bax* ^−/−^ mice was significantly elevated compared to preglaucomatous DBA/2J mice (*p* < 0.001). The width of the horizontal line (black in [A], gray in [B]) represents the mean IOP ± SEM of a group of wild-type preglaucomatous DBA/2J mice that were 3 mo old. The degree of IOP elevation, however, was altered in *Bax* ^−/−^ mice. In both 9- and 10.5-mo-old *Bax* ^−/−^ mice, the average IOP was less than that of *Bax* ^+/+^ and *Bax* ^+/−^ mice. This reduction in IOP elevation was significant at 10.5 mo (*p* < 0.01). The IOP of *Bax* ^+/−^ mice did not differ from wild type at either 9 or 10.5 mo (*p* > 0.82). By 12 mo, there was no difference in IOP between mice of any genotype (*p* > 0.25).

Although iris damage was similar in mice of all three genotypes, *Bax* genotype did have an effect on IOP. The peak period of IOP elevation in DBA/2J mice is from 9 to 12 mo of age, with the IOP distribution clearly shifting upward between 8 and 9 mo. We monitored IOP at key ages (9, 10.5, and 12 mo). Surprisingly, *Bax* ^−/−^ mice tended to have lower IOP than either *Bax* ^+/−^ or *Bax* ^+/+^ mice at 9 mo, and the difference was statistically significant at 10.5 mo of age (Figure 4). In contrast, the IOPs of *Bax* ^+/−^ and *Bax* ^+/+^ mice were not different. Because of the lower IOP in *Bax* ^−/−^ mice, we analyzed IOP in 4-mo-old mice of each genotype and found no differences (average ± SEM, number of eyes examined: *Bax* ^+/+^, 13.54 ± 0.45 mm Hg, *n* = 22; *Bax* ^+/−^, 13.77 ± 0.33, *n* = 22; *Bax* ^−/−^ 13.46 ± 0.34, *n* = 20; *p* > 0.5). This result indicates that BAX deficiency does not alter baseline IOP but does have an effect as the IOP increases to glaucomatous levels in older mice.

The lower IOP insult in *Bax* ^−/−^ mice does not account for the survival of their RGCs. This conclusion is supported by the normal RGC numbers remaining in *Bax* ^+/−^ mice with indistinguishable IOP from *Bax* ^+/+^ mice. Previous studies have shown that BAX deficiency allows RGC survival following axotomy or optic nerve crush [13]. By contrast, even when neuroprotective treatments are administered, only a small number RGCs survive in the short term (4–6 wk) in *Bax* ^+/+^ mice exposed to severe axon trauma [51,52]. Thus, there is no reasonable explanation for the finding of prolonged survival of RGCs that have no axons other than that BAX is a necessary RGC-intrinsic molecule for apoptosis in this glaucoma model.

### *Bax* Deficiency Delays Axon Degeneration

**Figure 5:**
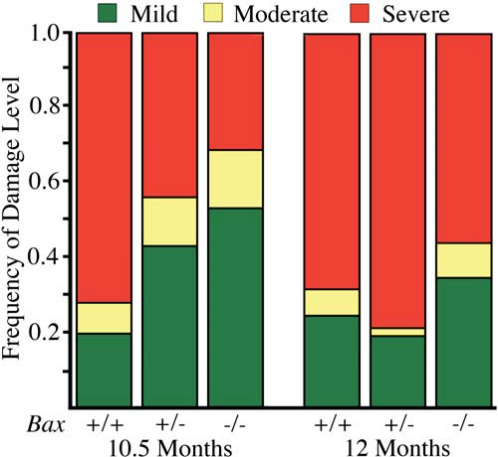
Glaucomatous Optic Nerve Degeneration Is Delayed in *Bax*-Deficient Mice BAX deficiency did not prevent glaucomatous axon degeneration. Nevertheless, the beneficial effect on RGC survival raised the possibility that it may have a protective effect on the axon and delay optic nerve damage. To assess this, three investigators masked to genotype determined the severity of optic nerve damage for mice of each *Bax* genotype at each age (*n* = 49–71 per genotype at each age; see Materials and Methods). At 10.5 mo, both *Bax* ^+/−^ and *Bax* ^−/−^ mice had significantly less optic nerve damage than *Bax^+/+^* mice. Mild damage was evident in 53% of *Bax* ^−/−^ and 44% of *Bax* ^+/−^ optic nerves, compared to only 20% of *Bax* ^+/+^ (*p* < 0.001 for both *Bax* ^+/−^ and *Bax* ^−/−^ compared to *Bax* ^+/+^; Chi^2^ test). With disease progression to 12 mo of age, the distribution of optic nerve damage became indistinguishable among mice of different *Bax* genotypes (*p* > 0.10). Quantitative assessment of a random subset of nerves assigned each damage level (more than eight of each) demonstrates that the number of axons that remain in optic nerves having each damage level are clearly different (see Materials and Methods).

Although we have shown that axon degeneration is not dependent upon BAX, our results clearly identify BAX as an endogenous susceptibility factor for both RGC death and axonal degeneration in DBA/2J glaucoma. As discussed above, complete or partial BAX deficiency had a profound rescuing effect on RGC cell bodies. Importantly, decreasing functional *Bax* gene dosage also decreased susceptibility to glaucoma by delaying the progression of axon damage (Figure 5). At 10.5 mo of age, the majority of *Bax* ^+/+^ mice had moderate or severe optic nerve damage (see Materials and Methods), with only 20% being mildly affected. In contrast, 53% of *Bax* ^−/−^ and 44% of *Bax* ^+/−^ mice were only mildly affected at 10.5 mo of age. At 12.0 mo of age, the distribution of optic nerve damage was indistinguishable among mice of the three *Bax* genotypes (Figure 5). Since mice of each *Bax* genotype were littermates that were housed in the same cages throughout aging, these results provide compelling evidence that decreasing BAX levels delays optic nerve damage.

The delay of optic nerve damage in *Bax* ^+/−^ mice (note: *Bax* ^+/−^ mice had similar IOP insults to *Bax* ^+/+^ mice) suggests that partially decreasing BAX levels in RGCs protects RGC axons. However, since complete BAX deficiency limited IOP elevation, a further protective effect of BAX deficiency by lowering IOP is also possible and may explain the trend toward greater axonal protection in *Bax* ^−/−^ mice. Thus, it is possible that either low-expressing or low-activity alleles of *BAX* may affect glaucoma susceptibility both by limiting and/or delaying IOP elevation and by directly protecting RGCs from damaging effects of harmfully high IOP.

### An Integrated Approach Supports a Role of Direct Optic Nerve Injury in Glaucoma

**Figure 6:**
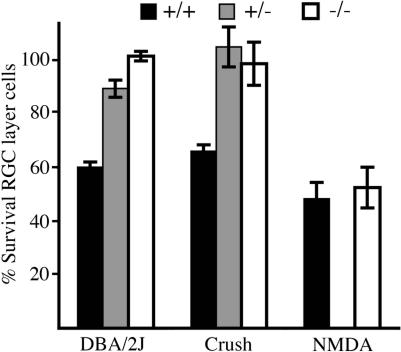
Mechanical Axon Insult, but Not Excitotoxicity, Induces BAX-Dependent RGC Death To help distinguish between the likely roles of mechanical axon insult and excitotoxicity in cell death induction in spontaneous glaucoma, we subjected preglaucomatous DBA/2J mice of each *Bax* genotype to either controlled optic nerve crush or NMDA-mediated excitotoxicity. For controlled crush and NMDA, the percent RGC survival in the manipulated eye compared to the contralateral control eye is shown. For ease of comparison, the data for glaucomatous damage are the same as shown in Figure 3. In contrast to the spontaneous glaucoma, NMDA-mediated RGC death is not dependent on BAX, as evident by the complete lack of protection from death in *Bax* ^−/−^ mice. As for the spontaneous glaucoma, RGC death induced by controlled optic nerve crush was completely dependent on BAX and prevented in both *Bax* ^+/−^ and *Bax* ^−/−^ mice. Overall, the effects of BAX in the face of spontaneous glaucoma and controlled crush were remarkably similar.

Comparing the specific pathways active in glaucomatous RGC death to the pathways induced by acute, experimental manipulations can provide information about the initial insult(s) to RGCs in glaucoma. *N*-methyl-D-aspartate (NMDA) receptor-mediated excitotoxic injury and direct axon injury are two insults that have been proposed to kill RGCs in glaucoma. Acute experimental procedures can be used to mimic these insults. Intraocular NMDA injection is used to mimic excitotoxic RGC insult, and controlled optic nerve crush is used to mimic a direct axon insult [53,54]. To assess the likely roles of these insults in a spontaneous glaucoma, we subjected preglaucomatous DBA/2J mice of differing *Bax* genotypes to these procedures. This allowed direct comparison of RGC death induced by these distinct excitotoxic and axonal insults to the naturally progressing glaucoma (Figure 6) in a single genetic context. *Bax* genotype had absolutely no effect on RGC death initiated by intraocular injection of the excitotoxin NMDA. In contrast, the RGCs of both *Bax* ^+/−^ and *Bax* ^−/−^ mice were profoundly protected against optic nerve crush. Since RGC death in the DBA/2J glaucoma is also BAX dependent, these data support a role for axon injury, but not for excitotoxicity (at least through the NMDA receptor) in this glaucomatous RGC death.

## Discussion

### BAX-Mediated Apoptosis Is Important in an Inherited Glaucoma

Our findings provide important new information about RGC injury and death in glaucoma. BAX deficiency completely prevents RGC death in DBA/2J mice. These results conclusively demonstrate that apoptosis plays a pivotal role in this inherited model of glaucoma. BAX is the first molecule shown to be completely necessary for RGC death in any glaucoma. Considering the protection we demonstrate in this mouse model, it is worth assessing BAX pathways as important targets for new treatments in human glaucoma.

### Distinct Pathways Mediate RGC Death and Axonal Degeneration in Glaucoma

Intrinsic axonal degeneration pathways have recently been identified [55,56]. The molecular components of these pathways appear to be distinct from those active in classical somal apoptosis [57,58]. Thus, the different compartments of a neuron can degenerate by different molecular processes. In glaucoma, it is not clear whether the same or different degeneration pathway(s) are activated in the cell body and axon. Our study demonstrates that BAX is required for RGC death but not for RGC axonal degeneration in DBA/2J glaucoma. This indicates that the axonal degeneration pathway is distinct from apoptosis in this inherited glaucoma. Our findings clearly demonstrate that axon degeneration is not a consequence of RGC death, since severe axon degeneration occurred in *Bax* ^−/−^ mice without RGC death. It is not yet clear whether the RGC apoptosis and axonal degeneration pathways have some common features or are completely distinct. However, for the design of therapeutic strategies for human glaucoma, our studies suggest that both apoptotic and axonal degeneration pathways should be considered.

### Alternative Glaucoma Hypotheses

The initial RGC compartments that are insulted in glaucoma, as well as the nature of the damaging insults that induce degeneration, are not completely clear. In the excitotoxic hypothesis of glaucoma, elevated IOP leads to elevated intraocular glutamate levels [59]. The elevated glutamate levels are proposed to cause excessive stimulation of glutamate receptors (NMDA type), leading to increased intracellular calcium levels and RGC death. A different glaucoma hypothesis involves direct optic nerve injury. In this hypothesis, high pressure places stress on the optic nerve as the nerve exits the eye through the lamina cribrosa [60]. Important studies report that the first damage to RGCs is evident in the axon segment near the lamina cribrosa in the optic nerve head [61,62], so it was suggested that this is the first site of IOP-induced insult (see Quigley [60]). Although it definitively shows local axonal dysfunction, the occurrence of initial damage in this region does not conclusively indicate that this is the first or only site of neuronal insult. Because of optic nerve head architecture and the stress at the lamina cribrosa, it is conceivable that the axon segment at the lamina cribrosa may take substantial resources to maintain, especially when IOP is elevated. Somal stress may decrease available resources for axon maintenance and repair. Therefore, somal stress or damage may contribute to the abnormalities observed in the optic nerve head. As a group, *Bax* ^+/−^ mice had an indistinguishable IOP insult compared to *Bax* ^+/+^ mice, but their RGCs did not undergo pressure-induced cell death. Importantly, RGC axonal degeneration was delayed in these *Bax* ^+/−^ mice. Therefore, our data imply that shielding the RGC cell bodies has a protective effect against axon degeneration.

### Direct Optic Nerve Damage Resembles Glaucoma

To provide insight to the nature and location of the damaging insults that occur in glaucoma, we compared the effects of BAX deficiency on RGC death in inherited glaucoma to RGC death induced by either direct optic nerve injury or excitotoxicity (all in the genetically uniform DBA/2J strain). Intraocular NMDA injection was used to model excitotoxic RGC death, and controlled optic nerve crush was used to mimic direct optic nerve damage [53,54]. Unlike the DBA/2J glaucoma, our experiments show that the excitotoxic insult does not require BAX to induce RGC death. Although these experiments cannot rule out the possibility of an intrinsic excitotoxic mechanism, these results do not support a role of NMDA receptor-mediated excitotoxicity as a primary cause of glaucomatous RGC death. Similar to the DBA/2J glaucoma, RGC death following optic nerve crush requires BAX, and both *Bax* ^+/−^ and *Bax* ^−/−^ mice are profoundly protected. Along with our demonstration of an axon intrinsic degeneration pathway, these results further support the hypothesis [60] that direct optic nerve and axon injury is an important pathogenic component leading to RGC death in glaucoma.

### *Bax* Can Modulate Neuronal Susceptibility in Glaucoma

Individual patients have different levels of susceptibility to glaucomatous RGC death [2,63]. Our experiments clearly identify *Bax* as an important modulator of neuronal susceptibility in DBA/2J glaucoma. BAX deficiency prevented RGC death and delayed optic nerve degeneration in both *Bax* ^+/−^ and *Bax* ^−/−^ mice. These results suggest that the use of BAX inhibitors could potentially be used to delay glaucomatous vision loss. In situations where BAX is important, pharmacologically suppressing BAX activity may significantly slow the progression of glaucoma. Since RGCs were maintained for an extended period after axon degeneration in *Bax* ^−/−^ mice, treatments that inhibit BAX pathways may allow long-term preservation of RGC cell bodies. Such treatments may allow the RGCs of patients to be stored in their own retinas until future treatment strategies are developed that can stimulate axonal growth and restore vision.

### Complete *Bax* Deficiency Limits IOP Elevation

In addition to implicating BAX as a target for direct neuroprotective treatments, the lower IOP of *Bax* ^−/−^ mice suggests that BAX inhibition may delay or limit IOP elevation. These results suggest that apoptotic death of cells affecting aqueous humor drainage contributes to IOP elevation, at least in secondary glaucomas where the drainage structures are insulted by pigment and cell debris. In a previous study assessing neuroprotection by an apoptosis inhibitor in a rat model of glaucoma, the treated rats had lower IOP than the other group [25]. Although not a conclusion of this rat study, the IOP data support a role for apoptosis in IOP elevation. In humans, cell death has been speculated to contribute to common forms of glaucoma (due to loss of drainage structure cells in old individuals and at late stages of glaucoma [64,65]). However, a primary role for ocular drainage pathway cell death during IOP elevation is not clearly established. Importantly, a recent study convincingly demonstrated endoplasmic reticulum stress and subsequent cell death in primary cultures of drainage pathway cells expressing human glaucoma mutations [66]. Together with our finding that complete BAX deficiency delays IOP elevation in a glaucoma setting, these results strongly support further investigation of apoptotic pathways and effects of antiapoptotic drugs on IOP in human glaucoma.

### *BAX* Is a Candidate Human Glaucoma Susceptibility Gene

The profound protection against RGC death and the delay in axon degeneration in *Bax* ^+/−^ mice together suggest *BAX* as a candidate human glaucoma susceptibility gene. It is important to note that we considered the possibility that a closely linked gene that was transferred from the 129/SV strain (in which the *Bax* mutation was generated) hitchhiked into the DBA/2J background along with *Bax* and explains the protection in heterozygotes. We conclude that this possibility is remote on the basis of the following observations. First, the RGCs of wild-type mice of the parental 129/SV strain are not protected from optic nerve crush. *Bax* heterozygosity protected the animals from both optic nerve crush and glaucoma in our experiments. This strongly implies that the parental strain does not have a modifier gene that would account for the protection we observed. Second, almost all RGCs were saved in the *Bax* ^+/−^ mice despite complete axon degeneration. To our knowledge, only two genes have been documented that can save the cell when the axon is destroyed. Substantial overexpression of *Bcl2* (a BAX antagonist) can do this, as can *Bax* deficiency. Thus, it is very unlikely that there is a similarly potent gene in the congenic interval, and scanning the flanking chromosome identifies no obvious candidates.

Complete BAX deficiency has developmental consequences [44] and is unlikely to be common in the human population. However, human *BAX* alleles that quantitatively affect the level of BAX are identified, and are reported to affect the development and progression of some but not other diseases [67–72]. Other factors that control *BAX* expression could also be important. Lower levels of BAX are associated with a worse prognosis for some types of cancer [73]. Our findings in *Bax* ^+/−^ mice support the hypothesis that quantitative variation in the level of *BAX* gene product may alter the prognosis of glaucomatous damage in individuals with high IOP. Although further studies are needed to assess this possibility, quantitative variation of BAX activity among human patients may have a substantial effect on susceptibility and disease progression. It is possible that lower-activity alleles may result in slower or less severe damage, whereas high-activity alleles may be detrimental. Characterization of *BAX* alleles may have important predictive value for disease progression.

## Materials and Methods

### 

#### Animals and husbandry.

Mice were housed in a 14 h light to 10 h dark cycle under previously described conditions [74]. The Jackson Laboratory (Bar Harbor, Maine, United States) pathogen surveillance program regularly screened for pathogens. All experiments were conducted in accordance with the Association for Research in Vision and Ophthalmology's statement on the use of animals in ophthalmic research and were approved by our institutional animal care and use committees. Both male and female mice were used. For each age group and genotype, approximately equal numbers of males and females were used. A *Bax* null allele (*Bax^tm1Sjk^* [44]; herein referred to as *Bax* ^−^) was backcrossed from B6.129X1-*Bax* ^*tm1SjK*^ (obtained from The Jackson Laboratory) onto DBA/2J for more than 12 generations to generate the congenic strain D2.129X1(B6)-*Bax* ^*tm1Sjk*^ */Sj*. Congenic DBA/2J *Bax* ^+/−^ mice were intercrossed to produce *Bax* ^+/+^ *,* *Bax* ^+/−^, and *Bax* ^−/−^ littermates. All three genotypes were housed together and analyzed simultaneously. DBA/2J mice were from our colony (Sj) that was initiated with mice purchased from The Jackson Laboratory. DBA/1J mice were obtained from The Jackson Laboratory.

#### Cell death related assays.

Eyes from DBA/2J or control DBA/1J mice were fixed in 4% paraformaldehyde in 0.1M phosphate buffer (pH 7.2) for 3 h, transferred to 0.4% paraformaldehyde in 0.1 M phosphate buffer for 48 h, and infiltrated with paraffin. Eyes from two 10- to 11-mo-old DBA/2J mice and two control mice were sectioned at 5 μm thickness and subjected to a modified double labeling protocol that involved in situ end-labeling (equivalent to a TUNEL assay) of fragmented DNA (using BODIPY fluorophores; Molecular Probes, Eugene, Oregon, United States) and detection of condensed chromatin (with the dimeric cyanine dye YOYO-1; Molecular Probes) as published [75]. Samples were analyzed with a confocal microscope. Conventional TUNEL assays were performed as previously reported [13] and conducted on the following numbers of DBA/2J mice of each age group: 7 mo (six), 8–9 mo (ten), 10–11 mo (16), 12–13 mo (nine), and 15–18 mo (eight). Five 10- to 12-mo-old control DBA/1J mice and more than 15 control mice of mixed genetic background ranging from 10 to 14 mo old were also analyzed. Counts of TUNEL positive cells were done as previously reported [53]. Briefly, the number of TUNEL-positive RGC layer cells was counted for eight to 12 sagittal sections from each eye, and average values for each age group are reported. Eyes used for electron microscopy and histology were processed as previously described [76], except that tissue blocks were oriented for en face retinal sectioning through the ganglion cell layer.

#### Clinical examination and intraocular pressure measurement.

DBA/2J mice develop a pigmentary form of glaucoma that follows a characteristic easily detectable clinical course. DBA/2J mice (all genotypes) used in the spontaneous glaucoma experiments were assessed with a slit lamp to ensure that the *Bax* mutation did not alter the course of the disease. Slit-lamp examination and evaluation criteria (including pigment dispersion and transillumination) were previously described [41,42]. Examination of at least 40 mice of each genotype at 6 and 9 mo of age and at the time of harvest (10.5 or 12 mo) was performed. Additionally, smaller groups of mice (12–20 of each genotype) were analyzed at other ages between 3 and 12 mo of age. IOP was recorded [77,78] for mice of each genotype. The number of mice of each genotype successfully assessed at each age were as follows. For 4 mo, *Bax* ^+/+^ *n* = 22, *Bax* ^+/−^ *n* = 22, *Bax* ^−/−^ *n* = 20; for 9 mo, *Bax* ^+/+^ *n* = 21, *Bax* ^+/−^ *n* = 25, *Bax* ^−/−^ *n* = 18; for 10.5 mo, *Bax* ^+/+^ *n* = 50, *Bax* ^+/−^ *n* = 54, *Bax* ^−/−^ *n* = 52; and for 12 mo, *Bax* ^+/+^ *n* = 42, *Bax* ^+/−^ *n* = 42, *Bax* ^−/−^ *n* = 37. Student's *t*-tests were used for statistical comparisons.

#### Optic nerve damage.

Optic nerves were dissected, processed, embedded in plastic, sectioned and stained with paraphenylenediamine (PPD) as previously described [76], except that the staining time was increased to 35 min and Embed 812 medium was used. PPD stains all myelin sheaths, but differentially stains the axoplasm of sick or dying axons darkly. Counts of normal-appearing axons were performed using established nonbiased counting methods. Prior to beginning axon counts, the optic nerve was outlined at 100× magnification, and its cross-sectional area was automatically calculated. Magnification of the same nerve section was increased to 1,000×, and a total of 20 fields at 1,000× were electronically collected. The fields were spaced in a regular fashion across the entire nerve, taking care to avoid field overlap so that the same area was not counted twice. The 20 collected pictures were stacked on the computer screen so that only the final picture was visible to the operator. For nerves with a large number of axons (mildly and moderately affected nerves), a rectangular box that contained a minimum of 200 axons was then drawn on the twentieth image. For nerves with severe axon loss, a larger box was drawn so that a significant proportion of the nerve could be counted. The software program then “cut” a rectangle centred at the same location in all 20 images. Since the operator could only see the top image, this removed the possibility of unconscious operator bias and made the selection of axons to be counted random. Axons were counted manually and marked using the computer. The program tracked the total area counted and the total axon count for all 20 images. The total counted area averaged 12.1%, 14.2%, and 20.5% of the total nerve area for mildly, moderately, and severly affected nerves, respectively. The final count was calculated and expressed as number of axons per optic nerve. With this approach, the nerves with 95% or more axon loss were selected for RGC counts by comparing the remaining axon number to the average for unaffected nerves of the same genotype.

Because of the large number of mice (approximately 50–70 mice of each genotype at each age), an optic nerve rating scale was used for the glaucoma progression study (see Figure 5). The indicated damage levels are readily distinguishable upon inspection of the nerve without counting. Nevertheless, axon counts were performed on at least eight randomly selected nerves of each damage grade to provide quantitative information about these distinct stages of disease (see below). Two investigators (masked to genotype, age, and the damage level assigned by the other investigator) assigned a damage level to each nerve. The two investigators assigned the same grade more than 90% of the time (321 out of 355 nerves). For the nerves on which the initial two investigators differed, a third (masked) investigator was utilized. The third investigator's grade always agreed with one of the initial grades, and the most common assigned grade was used. The number of nerves of each genotype assessed at each age were as follows. For 10.5 mo, *Bax* ^+/+^ *n* = 49, *Bax* ^+/−^ *n* = 62, *Bax* ^−/−^ *n* = 58; for 12 mo, *Bax* ^+/+^ *n* = 71, *Bax* ^+/−^ *n* = 50, *Bax* ^−/−^ *n* = 65.

The damage levels and typical numbers of normal axons present at each stage (determined through axon counts by an investigator masked to damage grade) follow. The representative axon counts were determined for randomly selected nerves of each grade using the counting procedure described above. In mildly affected nerves, there was very mild or no damage, with healthy axons having a clear axoplasm and intact myelin sheath (average number of axons ± SEM: 50,504 ± 1,988; *n* = 8). In moderately affected nerves, darkly stained, degenerating axons were readily detectable, but the vast majority of axons appeared completely normal (average number of axons ± SEM: 31,410 ± 2,199; *n* = 8 [79]). In severely affected nerves, there was extensive axon damage throughout the optic nerve with obvious axon loss (average number of axons ± SEM: 7,970 ± 2,150; *n* = 17). The axon number was significantly different between optic nerves of each damage level (*p* < 0.001 for all comparisons, *t*-tests).

#### Ganglion cell death.

Eyes were fixed and retinas were flat-mounted and Nissl-stained with cresyl violet using a modification of the technique reported by Stone [80]. Retinal ganglion cells make up approximately 40%–60% of the neurons in the ganglion cell layer of the mouse retina, and all RGC subtypes cannot be reliably distinguished from the other resident neuron in the ganglion cell layer (the displaced amacrine cell) based on cellular morphology [53,81]. This is especially true during disease, when morphology and marker expression can change dramatically. Consequently, cell loss was measured as a function of the change in total cell number compared to control eyes (strain and genotype matched nonglaucomatous eyes for the spontaneous glaucoma experiments and the contralateral nonmanipulated eye for the controlled crush and excitotoxic experiments). RGC density varies greatly with respect to retinal location. Therefore, two 40× fields were counted in each retinal quadrant and care was taken to ensure that the fields were the same distance from the periphery. For each individual eye, the eight counts for each retina were averaged. To assess RGC survival in the spontaneous glaucoma, retinas from eyes with very severely affected nerves that had fewer than 5% surviving axons were compared to retinas from unaffected eyes without glaucomatous nerve damage. RGC number was counted in approximately eight severely affected eyes and eight unaffected eyes of each genotype, except for unaffected control *Bax* ^+/−^ mice (five eyes) and 18 mo unaffected *Bax* ^−/−^ mice (four eyes).

#### NMDA injections and controlled optic nerve crush.

These experiments were performed as described previously [53]. For NMDA injections, 2 μl of an 80 mM solution of NMDA in balanced saline solution was injected intravitreally into one eye of each mouse using a glass micropipet. After 4 d the eyes were harvested and cells counted as described above. Data were collected from ten *Bax* ^+/+^ and eight *Bax* ^−/−^ mice. For optic nerve crush, the nerve of one eye was exposed and clamped approximately 0.5 mm from the globe with self-closing jeweler's forceps for 4 s. Eyes were harvested 21 d after surgery and cells counted. Data were collected from nine *Bax* ^+/+^, nine *Bax* ^+/−^, and seven *Bax* ^−/−^ mice. In each paradigm, cell loss was measured relative to the cell number present in the control eye of each mouse examined.

## Supporting Information

### Accession Numbers

The GenBank (http://www.ncbi.nlm.nih.gov/) accession number for *Bax* is 12028.

## Abbreviations

BAX: BCL2-associated X protein
IOP: intraocular pressure
NMDA: N-methyl-D-aspartate
RGC: retinal ganglion cell
SEM: standard error of the mean
